# Embryonic heat conditioning induces paternal heredity of immunological cross- tolerance: coordinative role of CpG DNA methylation and miR-200a regulation

**DOI:** 10.3389/fimmu.2025.1487135

**Published:** 2025-02-07

**Authors:** Padma Malini Ravi, Tatiana Kisliouk, Shelly Druyan, Amit Haron, Mark A. Cline, Elizabeth R. Gilbert, Noam Meiri

**Affiliations:** ^1^ Institute of Animal Science, Agricultural Research Organization, Volcani Center, Rishon LeZiyyon, Israel; ^2^ School of Neuroscience, Virginia Tech, Blacksburg, VA, United States

**Keywords:** cross-tolerance, heat conditioning, chick, embryo, hypothalamus, DNA methylation, miR-200a

## Abstract

**Background:**

Enhancing an organism’s survival hinges on the development of balanced and adaptable stress response systems. While the initial stress-response set-points in the hypothalamus may be genetically determined, they are further influenced by epigenetic factors during embryonic development. A debate persists regarding the heritability of such behavioral traits. The chick *in ovo* heat conditioning model offers a unique insight into this fundamental question, where manipulation during embryonic development can induce heat resilience and even cross-tolerance to promote immunological resilience. In this study, we conducted an analysis of thermal manipulation during embryogenesis to demonstrate paternal heredity and investigate its transmission through sperm DNA methylation in coordination with miR-200a action.

**Result:**

First-generation embryos underwent *in ovo* heat conditioning (EHC), creating a cohort of embryonic EHC and control chicks. These chicks were then subjected to an intracranial lipopolysaccharide (LPS) challenge. Conditioning rendered the chicks immune resilient, as evidenced by their fibril effect. Male offspring were raised to maturity, and their sperm was analyzed for methylome patterns, revealing significant differences between treatments, particularly in immune and development related genes. Additionally, sperm from EHC males was used for artificial insemination of naïve Cobb hens, resulting in untreated offspring that displayed immune resilience upon LPS challenge, indicating transgenerational effects. Overlap analysis of sperm methylome and differentially methylated sites (DMS) of offspring hypothalamus revealed inheritance of altered methylation associated with specific genes. Several of these genes are potential effectors of miR-200a, whose expression profile in the hypothalamus during LPS challenge was conserved across both generations. To evaluate the role of miR-200a in cross-tolerance acquisition, miR-200a was intracranially injected, and RNA-seq analysis of the hypothalamus revealed genes involved in the regulation of developmental and metabolic processes, stress, and immune response.

**Conclusion:**

This study demonstrates paternal trait heredity by revealing that EHC induces cross-tolerance with the immunological system, rendering chicks resilient to LPS that transgenerationally transmit this to untreated offspring. Additionally, analysis of sperm methylation patterns in EHC mature chicks led to identification of genes associated with neuronal development and immune response, indicating potential neural network reorganization. Finally, miR-200a emerges as a regulator potentially involved in mediating the cross-tolerance effect.

## 1 Introduction

Improving an organism’s chances of survival relies on developing balanced and adaptable stress response systems (1). This pivotal process is profoundly impacted by the embryonic environment, which plays a crucial role in sculpting neural circuits that establish the stress response set-point (2). In each individual, there is probably an initial stress-response set-point which is determined genetically. Nevertheless, the interaction of genetic and epigenetic factors during embryonic development further shapes the stress response systems (3, 4).

To unravel the mechanisms underlying the life-long and even heritable effects of stress during embryonic development, an effective research model is essential. Manipulating mammalian embryos presents challenges, often relying on maternal factors such as maternal depression or anxiety. In contrast, the avian chick offers an ideal candidate. With the ease of environmental manipulation, and susceptibility to pharmacological interventions, chicks provide an optimal system for studying developmental responses to environmental stressors (5). Furthermore, the similarity of their hypothalamic thermal control system to that of mammals, particularly in the preoptic anterior hypothalamus (PO/AH), further enhances their suitability for in depth study.

In recent years, both our team and others have led the way in utilizing the chick model to explore responses to embryonic and early-life thermal stress (6–9). Heat stress occurs when an animal’s thermoregulatory mechanisms falter, leading to a rise in body temperature beyond the thermal neutral zone (10). Thermosensitive neurons, crucial in this process, reside within the preoptic region of the hypothalamus (PO/AH). Studies by Piestun et al. (2008) in chicks revealed that thermal manipulation of eggs induces adaptations necessary for long-term regulation of body temperature (11). Additionally, research by Carvalho et al. (2020) and Loyau et al. (2016) highlighted the positive effects of embryonic heat conditioning (EHC) on body temperature (Tb), bodyweight (BW), and the immune response in post-hatch broiler chickens and quail (12, 13). Furthermore (9), demonstrated that EHC contributes to thermal and immune cross-tolerance through multilevel epigenetic changes in the hypothalamus of chickens. Consequently, these epigenetic effects could extend to impact the innate immune system of chickens in subsequent generations (14).

Activation of the innate immune system is suggested to trigger polyspecific resistance through epigenetic changes, which have been observed to exert long-term effects across vertebrates. For instance, mild heat stress in avian embryos can lead to the upregulation of Hsp70 and IL-6, resulting in stress tolerance and partial protection against apoptosis (15). Modified incubation temperatures in ducklings stimulated the immune system, indicating that incubation temperature could influence the immune response post-hatch (16). Furthermore, lipopolysaccharide (LPS) induces inflammatory and immune-stimulatory responses, activating signal transduction pathways that lead to the transcription of genes encoding factors involved in the inflammatory process, including interleukin-6 (IL-6), nuclear factor-κB (NF-κB), and enzymes that catalyze nitric oxide (NO) production (17, 18). Remarkably, the immune response to heat stress also enhances immunological responses to infections.

The debate surrounding the heritability of behavioral traits persists, and *in ovo* heat conditioning provides a unique window into this fundamental question. While the life-long effects of the embryonic environment are clear and apparent, the mechanisms underlying the adjustment of the set-point and its epigenetic inheritance are only starting to be revealed. Transgenerational effects might be mediated by epigenetic changes such as DNA methylation, histone modifications, and small non-coding RNAs (19). Interestingly, non-coding RNAs (ncRNAs) also regulate gene expression through various epigenetic mechanisms. Gene silencing by recruiting histone and DNA methyltransferase enzymes is the most reported epigenetic mechanism of ncRNAs. Small RNAs could serve as signaling molecules that transfer information between tissues and even across generations. Several studies have shown that Small non-coding RNAs (sncRNAs) such as PIWI-interacting RNAs (piRNAs), small-interfering RNAs (siRNAs), and microRNAs (miRNAs) are possible mediators in the transmission of environmental information through sperm cells (20). Recent advances in transcriptomic screening has facilitated the understanding of regulatory machinery of thermal stress at the level of microRNA.

miRNAs, approximately 22 nucleotides long, are involved in RNA silencing and posttranscriptional regulation of gene expression, through RNA-induced silencing complexes (RISCs) or microRNA ribonucleoprotein complexes (miRNPs) (21). They also regulate DNA methylation and histone modifications by targeting the enzymes responsible for these functions. We have previously shown that miR-138 inhibits *EZH2* expression in the chick PO/AH, resulted in decreases in histone H3 methylation at lysine 27 (H3K27) in response to environmental heat stress (22). In addition, research by Sengar et al. (2018) in livestock, indicates that miRNAs play regulatory roles during high environmental temperatures by targeting genes associated with stress responses. Modulating the expression of miRNAs can directly affect heat shock proteins (HSPs), immune, apoptosis, and inflammation-related genes, influencing cell survival under stress conditions (23).

The molecular mechanisms underlying transgenerational immune responses, specifically the regulatory role of miRNA in coordination with DNA methylation in sperm, remain poorly understood. The present study analyses the biological role of miR-200a in paternal transgenerational EHC cross tolerance with the immune system.

## 2 Methods

### 2.1 Experimental model and subject details

Cobb chicks, representing first-generation embryos, underwent *in ovo* heat conditioning. A cohort was established comprising EHC and control chicks, which were subjected to an LPS challenge. The remaining male offspring were raised to maturity, and their sperm was either analyzed for methylome patterns or used for artificial insemination of naïve Cobb hens. The resulting untreated offspring were subjected to an LPS challenge. Since methylome analysis of the sperm suggested a potential involvement of miR-200, miR-200a was intracranially injected, and the resulting body temperature measured and hypothalamic expression patterns analyzed using RNA-seq. All procedures conducted in this study were approved by the Volcani Center Animal Experimentation Ethics Committee and carried out in accordance with the guidelines set forth by the European Community Council.

### 2.2 Egg incubation in the first and second generation.

First generation fertile eggs of Cobb strain broiler (*Gallus gallus*) were obtained from Brown Hatcheries (Hod Hasharon, Israel) and incubated under either standard (Control) or embryonic heat conditioning (EHC) using two Type 65Hs automatic incubators (Massalles, Barcelona, Spain). In the first generation EHC, eggs were subjected to a temperature of 39.5°C and 65% relative humidity for 12 hours a day, from embryonic days 7 to 16. Control eggs were maintained at 37.8°C and 56% relative humidity throughout the entire 21-day incubation period. In the second generation, offspring of both treatments were raised under standard incubation conditions, i.e., at 37.8°C and 56% relative humidity (24).

#### 2.2.1 Bird housing

Hatched chicks were weighed, tagged and randomly divided into pens in climate controlled rooms at 32°C under 22/2 h cycle of artificial illumination with *ad libitum* access to food and water. Their sex was determined through wing feathering. On day 7 post hatch, the temperature was changed to the optimal brooding temperature of 30°C. Chicks were raised and bred according to the Cobb broiler management guide (COBB Broiler Management Guide) (25).

#### 2.2.2 LPS challenge

On day 10 post-hatch, both generations, were intracerebroventricularly (ICV) injected with either 0.3µg of LPS originating from *Salmonella* (Sigma, St. Louis, MO) or a 0.9% vehicle solution (0.9% NaCl). Body temperature of the chicks was measured, and they were sacrificed by decapitation 6 h post-injection (26).

### 2.3 Hypothalamic tissue collection, RNA extraction and cDNA synthesis

PO/AH (preoptic anterior hypothalamus) was dissected and immersed in RNA later pH 5.2 for total RNA and DNA isolation. Total RNA was extracted from the hypothalamus using TriReagent (Molecular Research Center, Cincinnati, OH, USA) according to the manufacturer’s protocol and checked for concentration and purity (260:280 nm absorbency). Hypothalamic RNA (0.5µg) was reverse transcribed to cDNA with SuperScript II Reverse Transcriptase and oligo (dT) plus random primers (Invitrogen, Carlsbad, CA, USA).

### 2.4 miR potential target identification

Online bioinformatics database miRDB (http://mirdb.org) was used to identify the potential targets of miR-200a in the chicken (*G. gallus*). Predicted targets from miRDB were compared with the F_1_ RRBS (S5 and S6) (https://faseb.onlinelibrary.wiley.com/action/downloadSupplement?doi=10.1096%2Ffj.202101948R&file=fsb222406-sup-0006-TableS5.pdf) (https://faseb.onlinelibrary.wiley.com/action/downloadSupplement?doi=10.1096%2Ffj.202101948R&file=fsb222406-sup-0007-TableS6.pdf) using interactive Venn (https://www.interactivenn.net/) to retain the common potential for miR-200a (9). The statistical significance for overlap was tested with the online program http://nemates.org/MA/progs/overlap_stats.html.

### 2.5 Real-time PCR

Real-time PCR was performed with 10 ng cDNA in a StepOnePlus Real Time PCR System (Applied Biosystems, Foster City, CA) with PerfeCta SYBR Green FastMix, ROX (Quanta BioSciences, Gaithersburg, MD, United States). Dissociation curves were analyzed following each real-time PCR to confirm the presence of only one product and the absence of primer-dimer formation. The threshold cycle number (Ct) for each tested gene (X) was used to quantify the relative abundance of that gene using the formula [2 ^(-Ct gene X –Ct standard)^]. Hydroxymethylbilane Synthase (HMBS) was used as the standard for mRNA expression. The primers used for real-time PCR were as follows: FCGTTTGGAGGGTGGCTGTAG; R-TGTCAAGTACAACTGGCCATCTTT (24).

miR-200a was quantified using TaqMan^®^ MicroRNA assays (Applied Biosystems) according to the manufacturer’s instructions. 10ng of total RNA was reverse transcribed using TaqMan^®^ MicroRNA Reverse Transcription Kit and miR-200a-specific RT-primer, followed by real-time PCR with TaqMan probes (Applied Biosystems). For each sample, relative miRNA expression was normalized to that of HMBS mRNA.

### 2.6 miR-200a mimic injection experiment

Hsa-miR-200a-3p mimic and miR-negative control (Thermo Fisher Scientific, Waltham, MA) were dissolved in saline (1µg/µl) and injected into the PO/AH of 3-day old chick (2.5 µg/chick). The expression of miR-200a was evaluated at various time points 0, 2, 6 or 24h using hsa-miR-200a Taqman Micro RNA assay (Applied Biosystems). Change in the body temperature was measured at 0, 2, 6 or 24h before decapitation and the PO/AH was dissected and immersed in RNA later.

### 2.7 RNA Sequencing and analysis

RNA-sequencing was performed at the Crown Genomics Institute of the Nancy and Stephen Grand Israel National Center for Personalized Medicine (INCPM), Weizmann Institute of Science (Rehovot, Israel) using the INCPM mRNA-seq protocol. Briefly, 15ng of purified RNA was used for library preparation with the bulk MARS-seq method, involving barcoding of samples by reverse transcription using an oligo dT primer, pooling of samples, and subsequent molecular reactions for linear amplification and preparation for Illumina sequencing (27). 100bp single reads were generated on a Novaseq 6000 sequencing system. The output was ~16 million reads per sample.

Poly-A/T stretches and Illumina adapters were trimmed from the reads using Cutadapt (28) and reads shorter than 30bp were discarded. The remaining reads were mapped onto 3′ UTR regions (1000 bases) of the Gallus genome (galGal5.0, UCSD) according to Refseq annotations, using STAR (29) with EndToEnd option and outFilterMismatchNoverLmax was set to 0.05. Deduplication was carried out by flagging all reads that mapped to the same gene and had the same UMI. Read counts per gene were quantified using HTSeq-count (30). UMI counts were corrected for saturation by considering the expected number of unique elements when sampling without replacement. Differentially expressed genes were identified using DESeq2 (31) with the betaPrior, cooksCutoff and Independent Filtering parameters set to False. Raw P values were adjusted for multiple testing using the procedure of Benjamini and Hochberg and the pipeline was run using snakemake. Respective heat maps were generated by custom R scripts and the Complex heatmap R package. Gene ontology (GO) analysis of upregulated and downregulated genes was performed using the online software Metascape (http://metascape.org).

### 2.8 Sperm collection, DNA isolation and reduced representation bisulfite sequencing and analysis

Sperm was collected individually and was stored immediately in -80°C. DNA was isolated from sperm samples using Genomic Mini AX Swab & Semen Spin (A&A Biotechnology, Gdansk, Poland) according to the manufacturer’s protocol. 70-140ng of DNA was used for RRBS library preparation using Zymo-Seq RRBS Library Kit (Zymo Research, Irvine, CA) at the Crown Genomics Institute of the Nancy and Stephen Grand Israel National Center for Personalized Medicine, Weizmann Institute of Science. Library quality was accessed through qubit (Thermo Fisher Scientific) and Tape Station (Agilent). Paired-end sequencing was done using a SP 100 cycle kit on Nova seq 6000 instrument (Illumina). Quality trimming and trimming of Illumina adapters was performed with Trim galore and the reads were mapped to the chicken genome (galGal 5.0) using Bismark v. 0-22-3 bowtie mode. Methylation calls were extracted with Bismark, in methylation extractor mode. Finally edge R (32) was used for extraction and analysis of differential methylation on single CpGs (DMS) with the cutoff of 8 reads in every sample and FDR <0.05. DMRs are extracted and analyzed by metilene v. 0.2-8 (33).

### 2.9 Targeted DNA methylation analysis

DNA was isolated from the same samples as RNA by TriReagent (Molecular Research Center, Cincinnati, OH, USA) according to the manufacturer’s protocol. Purified DNA (100 ng of each sample) was further processed for bisulfite modification using EZ DNA Methylation-Gold Kit (Zymo Research) according to the manufacturer’s instructions. We chose to evaluate the CpG methylation status between the control and EHC in the PO/AH within the miR-200a gene (NC_052552.1) region from - 61 to -230 bp relative to the transcription start site. Primers for this region were as follows: (5’→3’): miR-200a, F- GAGGAAATTGAGAAATTAAAGG, R- CCAACACTATCTAATAAAATACCC.

Amplified samples were sent for next-generation sequencing by the Crown Institute for Genomics at the Nancy and Stephen Grand Israel National Center for Personalized Medicine, Weizmann Institute of Science. Sequencing libraries were prepared using the INCPM DNA-Seq protocol (ChIP-Seq protocol). Long reads were sequenced on an Illumina MiSeq machine. The output was ~3.3033 (x10^4^) reads per library. Trimming of Illumina adapters, as well as quality trimming, were performed with Trimmomatic using the following options: -phred33, TruSeq3-PE.fa:2:30:10, SLIDINGWINDOW:4:15, LEADING:10, TRAILING:10 and MINLEN:36. Reads were mapped to the provided amplicon sequences using Bismark (version 0.13.1) with the options –bowtie2 and -nondirectional. Methylation calls were extracted using the MethylKit package (version 1.6.1). The Methylkit command processBismarkAln was used to read the aligned files using the option no lap set to TRUE. The average % C/T for each CpG per animal was calculated from the analysis, followed by the average of all animals per group.

### 2.10 Chromatin immunoprecipitation assays

To obtain fragments of 200–1000 bp, PO/AH tissues were crosslinked with 1% formaldehyde for 10 min and then sonicated in cell lysis buffer (1% SDS, 10 mM EDTA, and 50 mM Tris; pH 8.1) supplemented with protease inhibitor cocktail (Cell Signaling Technology, Beverly, MA) for 6 mins of 10 pulses on and off each using a Sonic-150w Ultrasonic Processor (MRC Ltd, Holon, Israel). Sheared chromatin samples were diluted in ChIP dilution buffer (0.01% SDS, 1.1% Triton X-100, 1.2 mM EDTA, 16.7 mM Tris-HCl, pH 8.1, 167 mM NaCl, and protease inhibitor cocktail) and incubated overnight at 4°C with antibody directed against NRF2 (4 µg/sample, Proteintech, Rosemont, IL) or mouse IgG as background IP (1 µg/sample; Merck Millipore, Darmstadt, Germany). Immunoprecipitates were separated by Magna ChIP Protein A+G magnetic beads (20µl/sample; Millipore) for 2 h at 4°C and reverse crosslinked in ChIP elution buffer [1% SDS, 100 mM NaHCO3, 0.2 M NaCl, and proteinase K (50µg/sample)] for 2 h at 62°C. DNA was isolated from each immunoprecipitate with Simple ChIP DNA Purification Buffers and Spin Columns (Cell Signaling Technology) and subjected to real-time PCR using miR-200a primer F- GGAGACGGGAACGAGGAAAC and R- AAAGATGGTGCAGGAAGTTGATG. Data were normalized to input samples that were not precipitated.

### 2.11 Statistical analysis

Statistical analyses for both the LPS challenge and miR expression profiles were performed using GraphPad Prism 6 software. Data normality was assessed using Bartlett’s test for variance equality and goodness of fit test. One-way analysis of variance (ANOVA) followed by Tukey’s multiple comparisons test or two-way ANOVA followed by Sidak’s multiple comparisons test were utilized for statistical analysis. The data presented in the figures are expressed as mean ± SEM. Various bioinformatics software packages were employed for statistical analysis of RNA-Seq, RRBS data and targeted methylation analysis, as detailed in the respective Materials and Methods sections.

## 3 Results

### 3.1 Paternal EHC prompts transgenerational immunological resilience in untreated offspring

**Figure 1:**
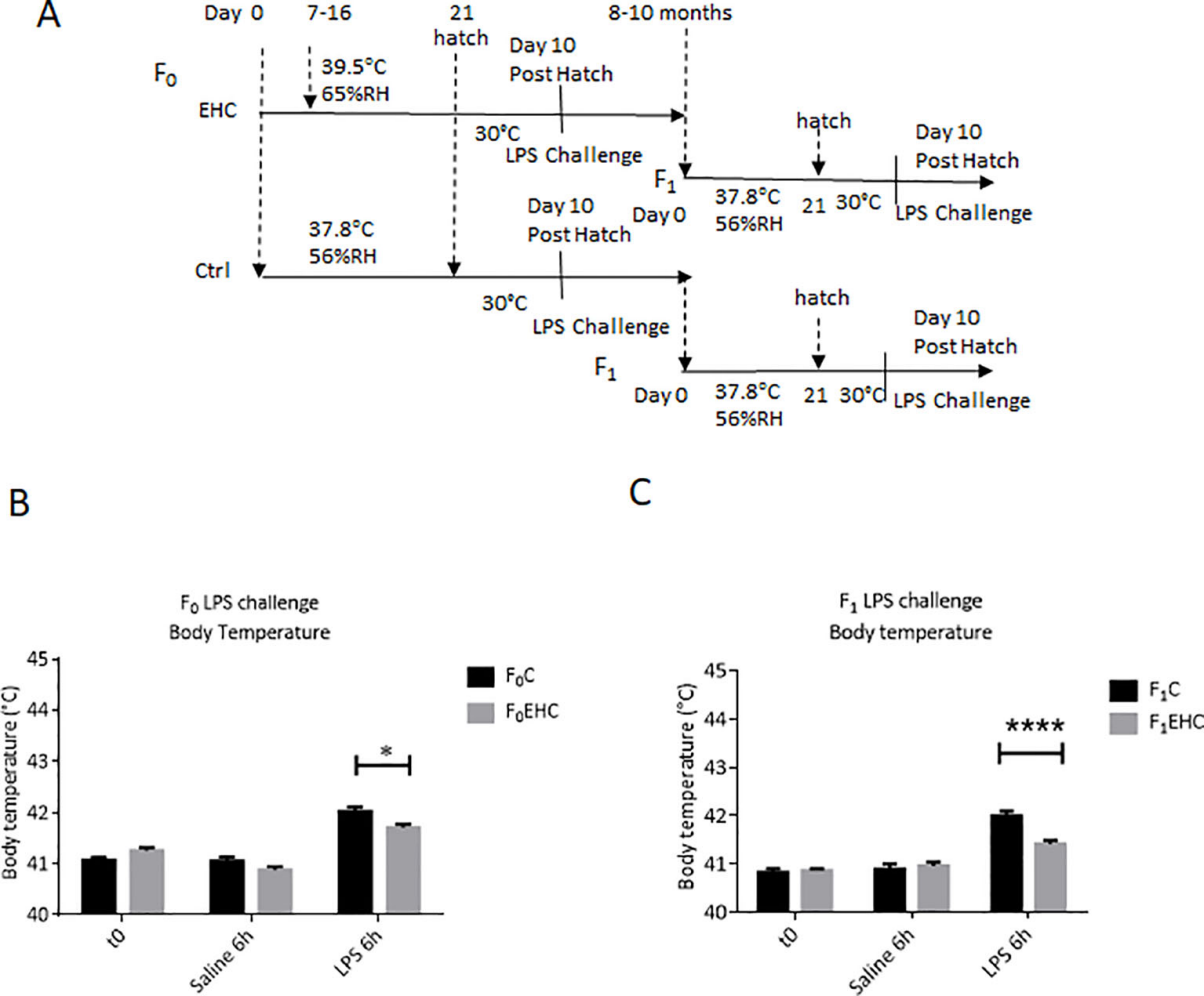
Paternal EHC prompts transgenerational immunological resilience in untreated offspring. **(A)**. Experimental design: EHC (F0) was performed during incubation on days 7-16 while control was maintained at standard incubation condition, then the animals were raised and bred to give offspring (F1). Both generations were challenged on day 10 posthatch with either 0.3µg of LPS or 0.9% saline into the PO/AH. **(B)** Body Temperatures of F0 were measured at time 0 (n: C=20; EHC=17); 6h after saline (n: C=20; EHC=17) and LPS challenge (n: C=20; EHC=17). **(C)** Body temperatures of F1 were measured at time 0 (n: C=14; EHC=24), 6h after saline (n: C=9; EHC=13) and LPS challenge (n: C=11; EHC=14). Data are represented as mean ± SEM. By Two way ANOVA test the significant effect is represented as 0.05 *p<0.01, ****p<0.0001.

The present study aimed to analyze the cross-tolerance impact of EHC on inflammatory resilience and to demonstrate its paternal heredity. To this end, a cohort from the first generation underwent EHC (F_0_). Both untreated and EHC male chicks were raised to sexual maturity and then bred with naïve hens to produce the F_1_ generation (**Figure 1A**). Ten days post-hatch in both generations, LPS challenge was performed to assess the immunological resilience by evaluating the fibril effect. LPS injection was effective, leading to a significant increase in body temperature for both F_0_ and F_1_ naïve chicks after 6 hours (p<0.01 for both comparisons; see **Figures 1B, C**). During the LPS challenge in both generations, the EHC-treated chicks had greater resilience to LPS and showed a smaller increase in body temperature. In the F_0_ generation, significant effects were observed in the interaction between EHC and control chicks (p=0.0017) and in the conditioning (p<0.0001) (**Figure 1B**). Sidak’s multiple comparisons showed a significant reduction in body temperature of F_0_EHC compared with F_0_C 6 hours after LPS injection (p=0.02) (**Figure 1B**). Despite being raised under identical conditions, the F_1_ offspring of EHC-treated fathers displayed greater resilience compared to offspring of fathers raised under standard conditions. The fibril effect of the increase in body temperature was significantly lower in the offspring of EHC-treated fathers, as indicated by differences in the interaction (p<0.0001), conditioning (p<0.0001), and LPS challenge (p=0.01) (**Figure 1C**). The injection itself did not induce any observable fibril effect, as evidenced by the comparison between the naïve chicks and those injected with saline (**Figures 1B, C**).

### 3.2 EHC alters CpG DNA methylation profile in sperm of F_0_ males

**Figure 2:**
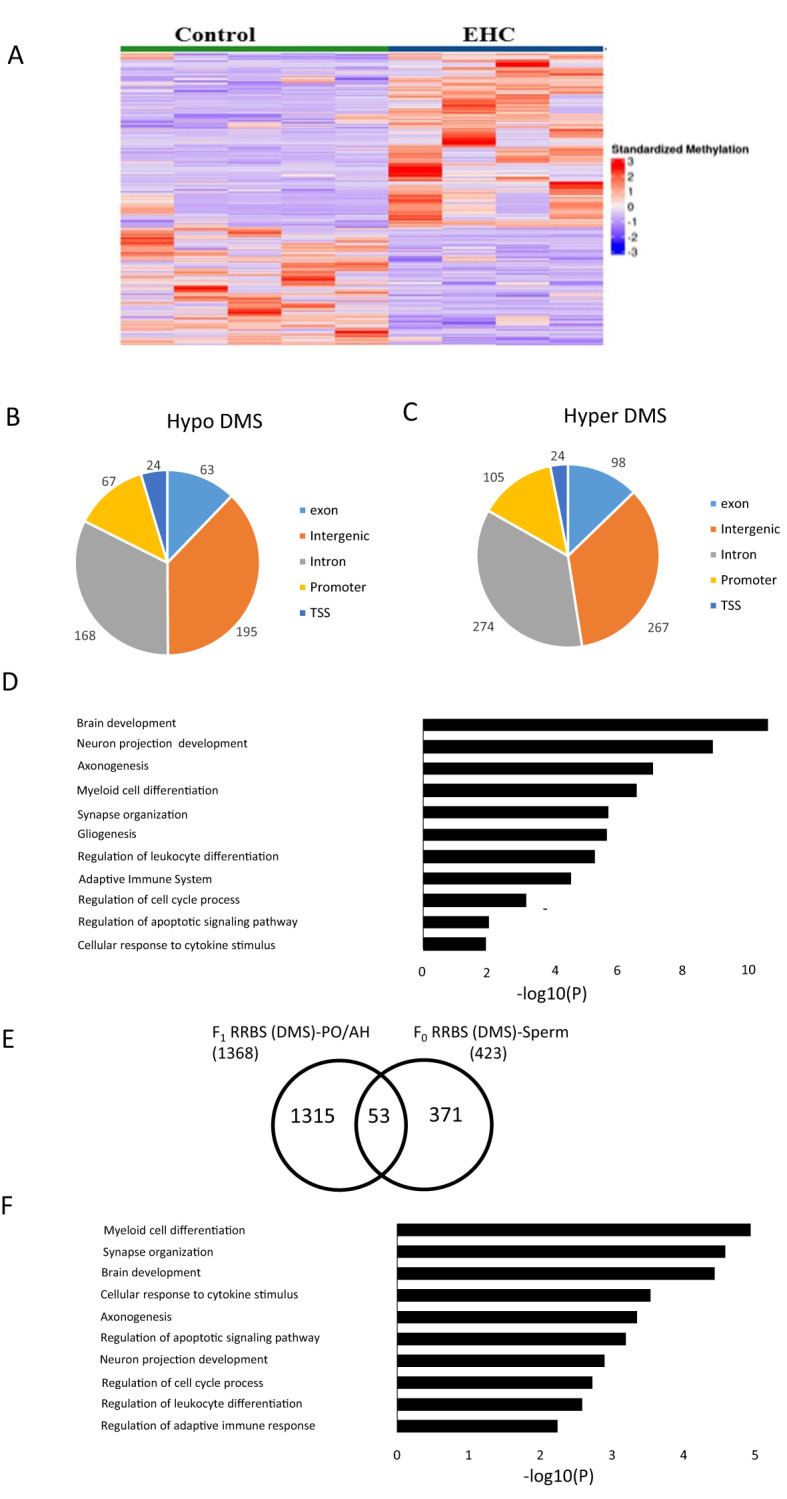
DNA methylation differences in response to EHC in F_0_ Sperm. **(A)** Heat map of significant DMS (1286) compared with control and EHC sperm. The samples are arranged as control and EHC. **(B)** Genomic distribution of hypomethylated-DMS shown in the pie chart includes exon, intergenic, intron, promoter and transcription start site **(C)** Genomic distribution of hypermethylated-DMS shown in the pie chart includes exon, intergenic, intron, promoter and transcription start site regions **(D)** GO analysis for the functional pathway of the hypomethylated and hypermethylated DMS. **(E)** Schematic representation of the DEGs which overlap between F_0_ RRBS Sperm and F_1_ RRBS PO/AH. **(F)** GO analysis for the common targets from F_0_ RRBS Sperm and F_1_ RRBS PO/AH.

To investigate the molecular mechanisms involved in transgenerational immunological resilience through F_0_ sperm, we conducted reduced representation bisulfite sequencing (RRBS) on DNA extracted from F_0_C and F_0_EHC sperm, analyzing their genome-wide DNA methylation patterns. Both DMSs and DMRs were analyzed and a list of the differentially methylated genes are presented as **Supplementary Files** (**Supplementary Tables S1**, **S2**). A heat map shows that the F_0_EHC sperm possesses a very significant difference in the methylation pattern of both hyper- and hypomethylated DMSs compared to the F_0_C ones (**Figure 2A**). Further analysis revealed a total of 1285 significant DMSs in which 768 are hyper DMSs and 517 hypo DMSs. The highest number of DMSs are in the intergenic (462) and intronic (442) regions followed by promoter (172), exon (161) and TSS (48) (**Figures 2B, C**). Although there are more differences in DMSs in the intergenic regions, this difference is likely due to the intergenic region being much larger than the promoter region, the occurrence of methylation is much higher in promoter regions. To better understand the gene networks and the biological processes that might be altered by differential methylation, gene ontology (GO) analysis was performed. Pathways with the strongest enrichment were found to be involved in different developmental aspects; from brain development to synapse organization and gliogenesis, immune cell differentiation and activation, cell cycle regulation and apoptotic process (**Figure 2D**). In addition to DMSs, sequence analysis displayed 99 DMRs between sperm of F_0_EHC chicks and their F_0_C counterparts (a list of differential methylated genes is presented in S2). Most DMRs found in F_0_EHC sperm are hypermethylated relative to that of F_0_C (74 hypermethylated and 25 hypomethylated DMRs). The distribution of hyper and hypo DMRs among the genomic regions occurred in intergenic (28) and intronic (26) regions followed by promoter (24), exon (9) and TSS (4).

To further understand the impact of EHC transgenerational inheritance on methylation patterns and gene expression from F_0_ sperm to F_1_ PO/AH, we compared the list of annotated genes with differential methylation (hyper/hypo DMSs) in F_0_ RRBS sperm to the previously published F_1_ RRBS PO/AH data (9). An overlap analysis identified 53 common genes (**Figure 2E**) with a significance level of p < 5.076 × 10^-5^. Gene ontology analysis of these genes shows their association with the pathways mentioned in **Figure 2D**, with the exception of gliogenesis (**Figure 2F**).

### 3.3 EHC influences miR-200a expression in both F_0_ and F_1_ generations.

Analysis of the F_0_ RRBS sperm data identified 21 miRNAs, predominantly located in intronic and intergenic regions, with only one miRNA found in an exonic region and two in promoter regions. We were particularly interested in identifying miRNAs involved in the transgenerational transfer of heat-stress resilience in coordination with CpG DNA-methylated genes in sperm. By literature survey, we selected miR-200a as a candidate for further study due to its known role as a regulator of signal transduction pathways related to cell cycle processes, growth, proliferation, differentiation, migration, and apoptosis (34, 35).

**Figure 3:**
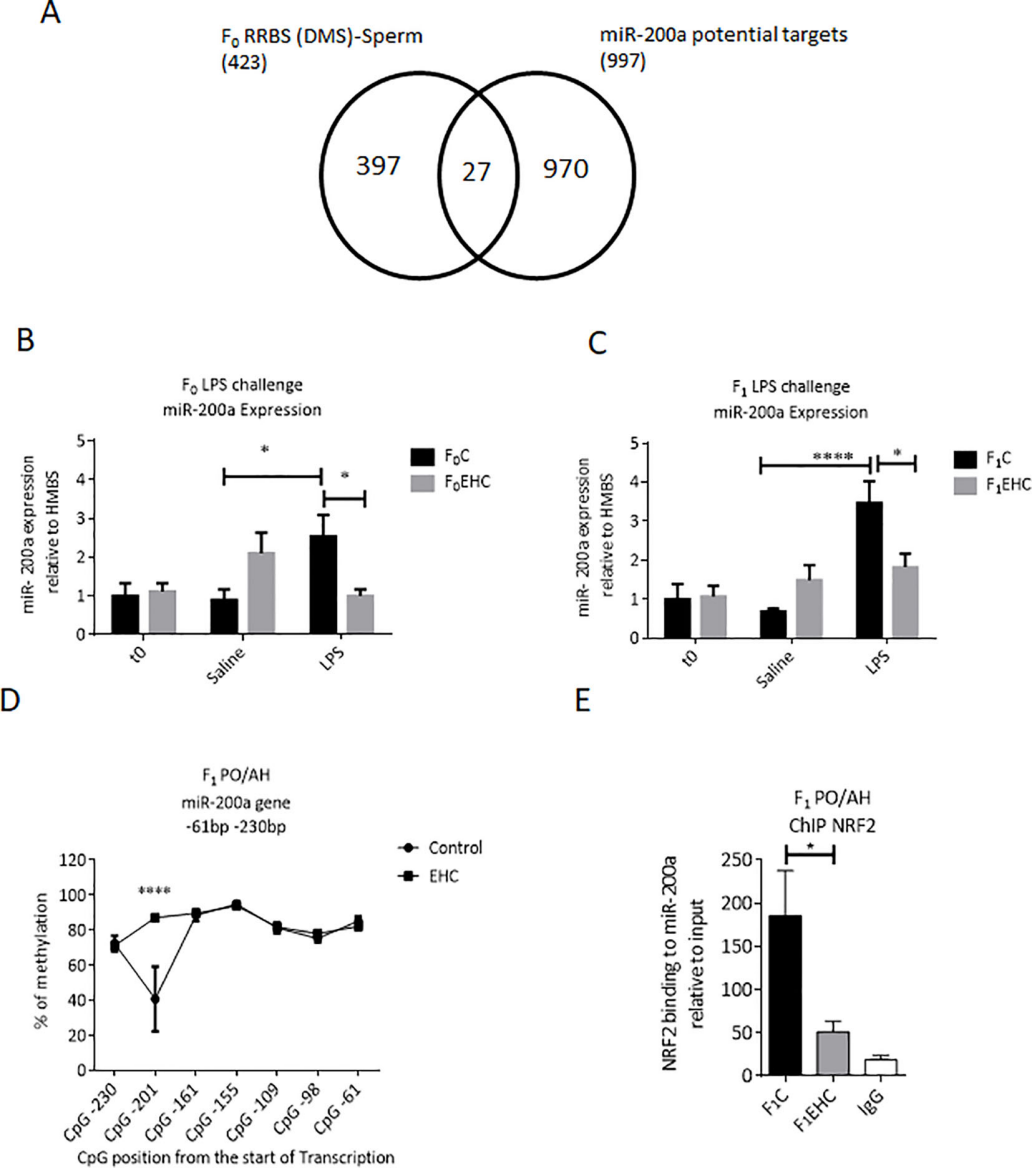
Expression of miR-200a in response to EHC in F_0_ and F_1_. **(A)** Schematic Venn diagram represents the computationally predicted targets of miR-200a-3p from miRDB compared with the F_0_ RRBS (DMSs)-Sperm. **(B)** Expression of miR-200a relative to HMBS in F_0_ (C and EHC) chicks at time 0 (n: C=10; EHC=9), 6h saline injected (n: C=12; EHC=10) or 6h of LPS challenge (n: C=12; EHC=13). **(C)** Expression of miR-200a relative to HMBS in F_1_ (C and EHC) chicks at time 0 (n: C=9; EHC=10), 6h saline injected (n: C=8; EHC=8) or 6h of LPS challenge (n: C=8; EHC=8). **(D)** C_p_G methylation of miR-200a between -61 and -230 bp from the start of transcription in PO/AH of Control and EHC. The C_p_G position from the start of the transcription start site are marked against the % of methylation on the y-axis. **(E)** ChIP of NRF2 binding to miR-200a relative to the input. Data are represented as mean±SEM. By Two way ANOVA test the significant effect is represented as 0.05 *p<0.01, ****p<0.0001.

The RRBS analysis of the sperm, we found a significant difference in methylation between control and EHC in a region on chromosome 21 *(chr21*: 2,589,560-2,592,022) which is located upstream of the miR-200a gene. Using the miRDB database (https://mirdb.org), miR-200a targets were predicted and overlapped with the differentially methylated genes (DMSs) detected by RRBS analysis of F_0_ sperm between control and EHC-treated embryos. MiR-200a had 27 targets in common with the differentially methylated genes of the total number of chicken genes (26,343) p < 0.006 (**Figure 3A**).

Responsiveness of miR-200a to EHC and its involvement in immunological cross-tolerance and paternal heredity, was assessed by expression profile in the PO/AH. Interestingly, expression of miR-200a in the PO/AH of control chicks (F_0_C and F_1_C) was significantly higher 6 hours after LPS injection compared to saline-injected counterparts (p=0.02 and p=0.001, respectively; **Figures 3B, C**). However, EHC attenuated the LPS effect on miR-200a expression in both generations (F_0_EHC and F_1_EHC); the level of miR-200a in the LPS-injected chicks was much lower than that in their control counterparts (p=0.04 for both groups) and remained similar to that of saline-injected ones (**Figures 3B, C**). The injection itself did not induce any observable change in the expression of miR-200a, as evidenced by the comparison between the naïve chicks and those injected with saline (**Figures 3B, C**). Thus, the attenuated response of miR-200a to LPS challenge in EHC chicks is transferred to the next generation.

We next aimed to examine the CpG methylation pattern of the miR-200a gene near the transcription start site in F_1_ PO/AH as a regulatory mechanism of miR-200a expression. Targeted methylation sequencing of the genomic region close to the transcription start site of the miR-200a gene (from -61 to -230 bp upstream of the transcription start site) in the F_1_ PO/AH found 7 unique CpG sites. Overall, the cluster of CpG nucleotides showed total methylation levels above 70% at most sites in both F_1_C and F_1_EHC chicks (**Figure 3D**). However, DNA methylation at CpG position -201 bp sharply reduced to 40% in the F_1_C group, while the methylation level of the same site in the F_1_EHC group was almost 90% (p<0.0001; **Figure 3D**).

*In silico* analysis of the miR-200a gene region from -61 to -230 bp upstream of the transcriptional start site using the TFBIND software program (http://tfbind.hgc.jp) identified numerous putative transcription factor-binding sites. Of these, nuclear factor-erythroid-2-related factor 2 (NRF2) was chosen for further analysis because it was predicted to bind with high affinity and close proximity to the CpG site at -201 bp upstream of the miR-200a transcription start. ChIP analysis was performed to examine NRF2 binding to the miR200a gene. As depicted in **Figure 3E**, NRF2 binding level to the analyzed region of the miR-200a gene was more than 3.5 times lower in the F_1_EHC group compared to the F_1_C group (p=0.01; **Figure 3E**). Immunoprecipitation with normal mouse IgG was used as a background control (**Figure 3E**). These results imply an inverse correlation between CpG methylation of the miR-200a gene region proximal to the transcription start, specifically at CpG position -201 bp, and the binding of the NRF2 transcription factor. This may result in differential expression of miR-200a in F_1_C and F_1_EHC in response to LPS challenge.

### 3.4 Intracranial injection of miR-200a alters gene expression patterns in the PO/AH

**Figure 4:**
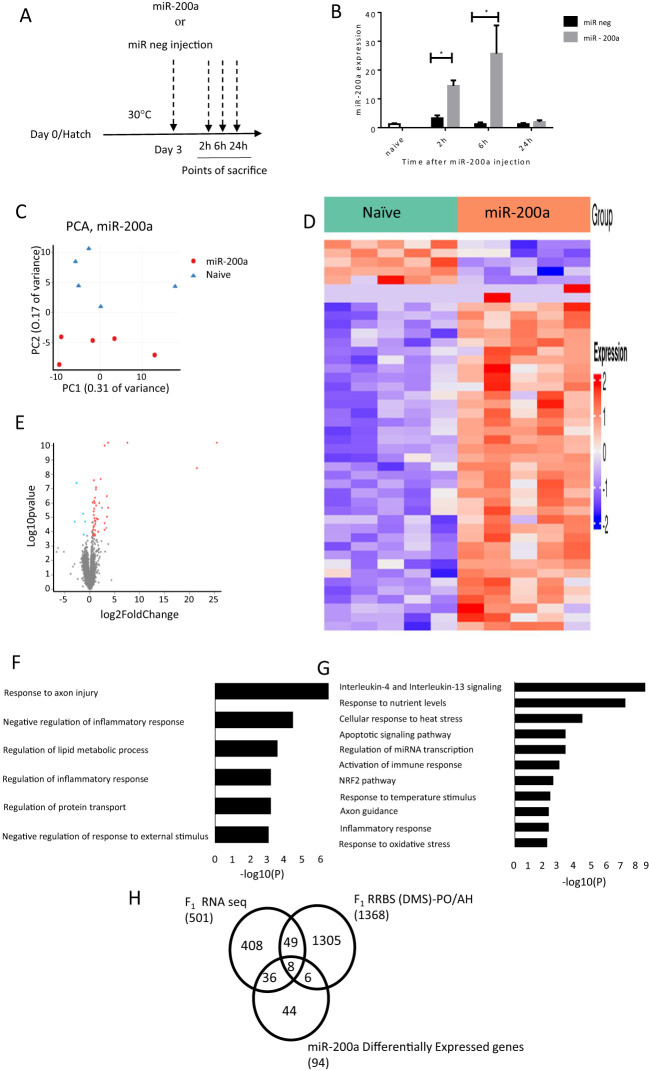
Differentially expressed genes after intracranial injection of miR-200a at 6h. **(A)** A schematic representation of the experimental design: miR mimic negative and hsa-miR-200a-3p mimic were injected into the PO/AH of 3 day old chicks (2.5 µg/chick) and compared to non-injected age matched naïve chicks. **(B)** Expression of miR-200a at 2, 6 and 24h after injection were quantified using a Taqman MicroRNA-200 assay (n=10 in each treatment/time point). **(C)** PCA of the gene expression of naïve and miR200a at 6h after intracranial injection. **(D)** Heat map for the significantly differentially expressed genes 6h after intracranial injection of naive and miR-200a. The gene sets are arranged based on the group and the color intensity (Blue-down and Red-up) represented by log2 FC of the expression. **(E)** Volcano plot of RNA-seq data in comparison between miR-200a vs naive. Fold Change in the gene expression is plotted in the x-axis against the log10 pvalue in the y-axis. The blue dot indicates the down regulated genes and the red dot indicates the upregulated genes. **(F)** GO for miR-200a down regulated genes. Bar length represents log10 (p-value) for the presented pathway. **(G)** GO for miR-200a Up regulated genes. Bar length represents log10 (pvalue) for the presented pathway. **(H)** Schematic representation of the DEGs which overlap between F_1_ RNA seq (LPS; control and EHC), F_1_ RRBS (DMS)-PO/AH and miR200a up and down regulated genes. Data are represented as mean±SEM. By Two way ANOVA test the significant effect is represented as 0.05 *p<0.01.

To explore the role of miR-200a in regulating gene expression in the PO/AH, a hsamiR-200a-3p mimic was intracranially injected into the third ventricle of 3-day-old chicks. Effectiveness of miR-200a incorporation was evaluated by measuring its levels in the PO/AH compared to those in counterparts injected with a mimic-negative control and naïve chicks at 2, 6, and 24 hours post-injection (**Figure 4A**). As depicted in **Figure 4B**, a 2.5-μg dose of miR-200a mimic was highly effective, with miR-200a levels in the PO/AH four times higher 2 hours post-injection compared to mimic-negative control-injected chicks (p=0.0001). The maximum amount of miR-200a, a 25-fold increase compared to mimic-negative control-injected chicks, was observed after 6 hours (p=0.02; **Figure 4B**). After 24 hours, the expression of miR-200a returned to the baseline level observed in mimic-negative control and naïve chicks (**Figure 4B**). The injection of miR-200a mimic did not affect chick body temperature (data not shown).

Given that the maximum amount of miR-200a in the PO/AH was observed 6h after miR-200a mimic injection, we conducted RNA-Seq analysis on samples extracted from the PO/AH of naïve chicks and those collected 6 h after miR-200a mimic injection.

Principal component analysis (PCA) revealed a substantial difference between naïve and miR-200a mimic treatment groups (PC1: 31% variance and PC2: 17% variance) (**Figure 4C**). The heat map displays the log_2_FoldChange values of differentially expressed genes (DEGs; padj ≤ 0.05), illustrating the expression levels of 44 genes altered following miR-200a mimic injection compared with naïve chicks (**Figure 4D**). The volcano plot depicts the 39 upregulated and 5 downregulated genes in the comparison between naïve and miR-200a-treated groups, based on the threshold for significant differential expression (padj ≤ 0.05, FoldChange 1.5, and min count ≥ 30) (**Figure 4E**). The list of upregulated and downregulated genes at padj ≤ 0.1 is presented as **Supplementary Tables S3** and **S4**, respectively. Gene ontology downregulated included Response to axon injury (FOLR1, CSPG5, TREM2), Negative regulation of inflammatory response and Negative regulation of response to external stimulus (LPCAT3, TREM2, LRFN5) (**Figure 4F**). Gene ontology analysis of the upregulated genes following 6 hours of miR-200a intracranial injection revealed associations with genes involved in Interleukin-4 and Interleukin-13 signaling (such as FOS, HSPA8, IL2RG, STAT3, STAT6, HSP90B1 and SOCS3), cellular response to heat stress (including HSPA2, HSPA5, HSPA8 and BAG3), activation of immune response (C1QA, C1QB, C1QC, C1S and NFKBIZ), NRF2 pathway (FTH1, GPX2 and SLC2A1), axon guidance (AP2S1, HSPA8, RPL30, ARPC1B and RPL35) and response to oxidative stress (FOS, GPX2, STAT6 and KLF2) (**Figure 4G**).

In order to better focus our understanding of miR-200a function, the genes that overlap between the F_1_ RNA seq, F_1_ RRBS and miR-200a effectors were overlaid. The Venn diagram represented in **Figure 4H** points to 36 common genes between F_1_ RNA seq and miR-200a regulated genes. Most of them involved in immune system activation (C1QA, C1QB, C1QC, C1S, TREM2, HSPA8, IRF1, SPI1, TAPBPL, and NFKBIZ) and to cellular response to heat stress (HSPA5, HSPA8 and BAG3).

The 8 genes common to the 3 comparisons i.e. F_1_ RNA-Seq, RRBS and genes affected by miR-200a are: STAT3, SLC2A1, IL2RG, HPDGS, HSPA2, OASL, FOS, TMEM141.

## 4 Discussion

Debate regarding the heritability of behavioral traits persists, and chick *in ovo* heat conditioning offers a unique perspective on this fundamental question. This study utilized an *in ovo* heat conditioning model to demonstrate paternal trait heredity by showing that EHC not only induces cross-tolerance with the immune system, making the chicks resilient to LPS, but also transgenerationally transmits this phenomenon to untreated offspring. Analysis of the methylation pattern of the sperm from mature chicks revealed a significant difference in the methylation pattern between chicks that underwent EHC and those incubated under standard conditions. Finally, miR-200a is suggested as a regulator involved in mediating the cross-tolerance effect.

In recent years, both our team and others have employed the chick model to explore responses to embryonic and early-life thermal stress (6, 11, 22, 36, 37). This model entails subjecting chick embryos to alternating high ambient temperatures between days 7 and 16, thereby inducing thermal resilience that persists throughout their lives. Additionally, *in ovo* heat conditioning of chick embryos triggers heat and immunological cross-tolerance by mitigating hypothalamic inflammation (9). These findings encompass changes in gene expression and their multilevel epigenetic modifications (38). Epigenetic modifications in genes such as BDNF and CRH contribute to the adaptation to high temperatures (6). To mimic inflammatory and infectious conditions, LPS is utilized as an immune stimulant (39). In this study, we demonstrated that EHC not only induces inflammatory resilience, as evidenced by the attenuated fibril response to LPS, but also that this phenomenon is paternally transmitted to offspring.

This study investigated heredity using the chick model of EHC via paternal transmission. This approach offers two distinct advantages: Firstly, in the F_1_ generation, the offspring from male parents of F_0_ were examined with EHC conducted from day 7 to 16, is the period when primordial germ cells (PGCs) are susceptible to conditioning effects, and when sperm cells, that develop later, and are not subjected to environmental manipulations. Secondly, this approach capitalizes on the ease of sequencing sperm to elucidate the mechanism of transgenerational transfer.

Since paternal inheritance of cross tolerance has been demonstrated, it suggests the involvement of a mechanism implicating sperm methylation. Analysis using RRBS revealed a significant difference in the methylation patterns between chicks that underwent EHC and those incubated under standard conditions. Notably, the genes potentially affected by these methylation differences are known to play roles in both brain and immune system development (**Figure 2F**). These pathways may indicate activation of immunological-related neuronal network reorganization, which could explain the inheritance of immune cross-tolerance phenomena. Changes in sperm methylation impact developmental progression and various traits. For instance, Keyhan et al. (2021) demonstrated that male obesity can significantly impact DNA methylation reprogramming in sperm (40). Additionally, Swinford-Jackson et al. (2022) found that cocaine-induced changes in sperm Cdkn1a methylation are linked to cocaine resistance in offspring (41). Moreover, Milekic et al. (2015) showed that age-related sperm DNA methylation changes can be transmitted to offspring and are associated with abnormal behavior and dysregulated gene expression (42).

To explore potential mechanisms underlying the transgenerational transmission of EHC-induced cross-tolerance immunological resilience traits, we sought a miRNA known for its involvement in immune regulation and whose targets are differentially methylated in the sperm between EHC and control male chicks. Previous studies by Sharma et al. (2023) and Shandilya et al. (2023) highlighted the roles of miR-200a and miR-141 in regulating the TLR4 gene, associated with various biological pathways in immune regulation and anti-inflammatory function (43, 44). Moreover, miR-200a-3p, is overexpressed in tissues with high levels of oxidative stress (45). Notably, thermal stress induces cellular oxidative stress, leading to the accumulation of free radicals and peroxides (46, 47). Studies have demonstrated that miR-200a-3p regulates inflammatory factors and genes related to the MAPK signaling pathway in response to Necrotic Enteritis in chickens (48). Therefore, it was of interest to explore a role of miR-200 in cross-tolerance acquisition.

Extraction of 27 miR-200a targets that were differentially methylated in the sperm between EHC and control chickens, found that EHC attenuated miR-200a expression in response to LPS in F0, and furthermore, this parental effect of EHC was transmitted to the offspring (F1) (**Figures 3B, C**). Additionally, we hypothesize that hypermethylation of specific CpG sites at the miR-200a gene, particularly -201bp upstream of the start of transcription, accompanied by a reduction in NRF2 binding, affects miR-200a expression in response to LPS challenge. NRF2 is a well-known regulator of antioxidant signaling and is involved in regulating brain physiology and the development of age-related neurodegenerative diseases (49). Thus, the CpG methylation pattern of the miR-200a gene may reflect EHC-related transgenerational epigenetic memory.

miRNAs serve as effective signaling regulators of genomic translation, modulating numerous targets through diverse regulatory pathways (50). The direct role of miR-200a in hypothalamic gene network rearrangement was demonstrated through direct intracranial injections it into the PO/AH with peak ectopic expression of miR-200a observed 6 hours after injection, (**Figure 4B**). The time point of 6 hours seems to be critical as employing a similar experimental protocol, revealed that intracranial injection of a different miRNA, specifically miR-138, effectively downregulated the expression of its target EZH2 at the 6-hour time point (51).

To delve deeper into the impact of intracranial miRNA-200a injection on the transcriptomic profile we conducted an RNA-Seq analysis. This analysis revealed that miR-200a exerted a notable influence on the hypothalamus transcriptome, resulting in the downregulation of 21 genes and upregulation of 73 genes. It is plausible that the downregulated genes represent a direct effect of miR-200a, while the upregulated genes may reflect an indirect pathway-mediated effect. Our initial focus was on elucidating the mechanisms underlying the downregulated genes. These downregulated genes, among others, include genes involved in response to axon injury (FOLR1, CSPG5, TREM2) and negative regulation of response to external stimulus (LPCAT3, TREM2, LRFN5). A study by Iskandar et al. (2010) shows that FOLR1 (Folate receptor R1 receptor), expression in a rodent model was increased with CNS injury leading to the uptake of folate which is then reduced to tetrahydrofolate (THF) by DHFR (dihydrofolate reductase). Methyl-THF is then required for the regeneration of SAM, which in turn promotes sufficient DNA methylation crucial for CNS regeneration (52).

Genes upregulated in the PO/AH and differentially affected by EHC, appear to be highly relevant to stress and immunological responses. Among them are members of the heat shock protein (HSP) family, including HSPA2, HSPA5, HSPA8, and HSP90B1, known for their roles in cellular stress responses (53). Additionally, transcription factors such as FOS, SOCS3, and STAT3, that are involved in mediating stress and immune responses, were also upregulated (9, 54, 55). It’s noteworthy that the HSPs and transcription factors mentioned above, along with SOCS3, were not only upregulated in the PO/AH of chicks injected with miR-200a but also exhibited differential expression under EHC treatment.

Finally, another noteworthy comparison includes the 8 genes encompassing the 3-way comparison between the genes differentially expressed in the PO/AH F_1_RNAseq, differentially methylated in the sperm, and those affected by miR-200a (**Figure 4H**). These 8 genes emphasize possible stress-related neural network reorganization during the EHC process. Among these genes emerges HSP2A, which is a heat shock protein belonging to the HSP70 gene family. We previously demonstrated a role for HSP70 in heat resilience with methyl CpG levels at its distal part exhibiting epigenetic memory for heat stress by modulating recruitment of POU2F1-associated nucleosome-remodeling deacetylase (NuRD) complex (22). Wang et al. (2020) showed miR-200a to exhibit anti-inflammatory and anti-atherosclerotic activity depending on the EZH2/STAT3 signaling cascade (56). In addition to stress related genes, the list includes immune-related genes such as interleukin 2 receptor subunit gamma (IL2RG) which is involved in regulation of tolerance and immunity (57).

This research highlights several unresolved questions that warrant further investigation. One area of uncertainty involves the specific cell types within the PO/AH that are affected by EHC and immunological cross-tolerance. Since our molecular analyses relied on tissue punches from the PO/AH, it remains unclear whether the observed effects are mediated by glial cells, neurons, or specific subpopulations of these cells. Identifying the precise cellular targets would provide valuable insights into the underlying mechanisms.

Another promising direction for future research involves exploring additional microRNAs (miRs) that may contribute to these processes. Although this study focused on miR-200a, demonstrating its involvement in heat and immunological cross-tolerance through correlative and interventional approaches, its definitive role in transgenerational inheritance has yet to be fully established. Further investigations could confirm its contribution and identify other miRs that may play complementary or independent roles in the observed phenomena.

Finally, a significant number of differentially methylated sites were identified in intergenic regions. These sites may influence regulatory elements, such as enhancers or repressors, which could play a key role in the transgenerational transmission of information. Exploring the functions of these intergenic regulatory elements represents an important step toward understanding the mechanisms underlying epigenetic inheritance.

## 5 Conclusion

These findings collectively underscore the critical importance of paternal EHC in nurturing both heat and immunological resilience in offspring. Furthermore, they corroborate the heredity of behavioral traits, shedding light on the role of parental influences in shaping offspring responses to environmental stressors. Moreover, the results suggest involvement of epigenetic mechanisms, such as sperm methylation and miRNA regulation, in mediating these transgenerational effects. These insights deepen our understanding of the complex interplay between environmental exposures, genetic inheritance, and epigenetic modifications in shaping offspring phenotypes and adaptive responses.

## Data Availability

The data presented in the study are deposited in the GEO repository. Accession numbers for RNA-Seq data: GSE282656 and for RRBS data: GSE282655.
